# Proteomics-based evaluation of AAV dystrophin gene therapy outcomes in *mdx* skeletal muscle

**DOI:** 10.1172/jci.insight.197759

**Published:** 2025-11-27

**Authors:** Erynn E. Johnson, Theodore R. Reyes, Jeffrey S. Chamberlain, James M. Ervasti, Hichem Tasfaout

**Affiliations:** 1 Department of Biochemistry, Molecular Biology and Biophysics, University of Minnesota - Twin Cities, Minneapolis, Minnesota, USA.; 2 Department of Neurology,; 3 Senator Paul D. Wellstone Muscular Dystrophy Specialized Research Center, and; 4 Department of Biochemistry, University of Washington School of Medicine, Seattle, Washington, USA.

**Keywords:** Genetics, Muscle biology, Biomarkers, Gene therapy, Proteomics

## Abstract

Duchenne muscular dystrophy (DMD) is a fatal genetic muscle-wasting disease characterized by loss of dystrophin protein. Therapeutic attempts to restore a functional copy of dystrophin to striated muscle are under active development, and many utilize adeno-associated viral (AAV) vectors. However, the limited cargo capacity of AAVs precludes delivery of full-length dystrophin, a 427 kDa protein, to target tissues. Recently, we developed a method to express large dystrophin constructs using the protein *trans*-splicing mechanism mediated by split inteins and myotropic AAV vectors. The efficacy of this approach to restore muscle function in *mdx*^4cv^ mice was previously assessed using histology, dystrophin immunolabeling, and Western blotting. Here, we expand our molecular characterization of dystrophin constructs with variable lengths using a mass spectrometry–based proteomics approach, providing insight into unique protein expression profiles in skeletal muscles of wild-type, dystrophic *mdx*^4cv^, and AAV-treated *mdx*^4cv^ mice. Our data reveal several affected cellular processes in *mdx*^4cv^ skeletal muscles with changes in the expression profiles of key proteins to muscle homeostasis, whereas successful expression of dystrophin constructs results in an intermediate to complete restoration. This study highlights several biomarkers that could be used in future preclinical or clinical studies to evaluate the effectiveness of therapeutic strategies.

## Abstract 2

This study describes a new method using proteomics to evaluate the efficacy of three different dystrophin replacement approaches using AAV vectors.

## Graphical abstract

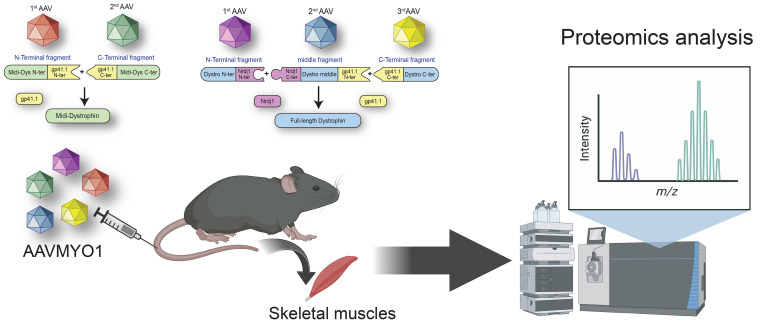

## Introduction

Duchenne muscular dystrophy (DMD) is an inherited, lethal X-linked muscle-wasting disease. Affected patients typically show the first symptoms at approximately 3 years of age (1). The muscle function deteriorates rapidly starting at the age of 7 years with pronounced muscle weakness, chronic inflammation, and fibrosis, leading to loss of ambulation and premature death in the second to the third decade of life due to cardiorespiratory complications. DMD is caused by loss-of-function mutations in the *DMD* gene that abolish the production of a functional dystrophin (2–4). In muscle, dystrophin constitutes a key partner to several proteins, which together form the dystrophin-glycoprotein complex (DGC) (5). This complex plays an important role in preserving myofiber integrity during muscle contraction by connecting the intracellular cytoskeleton to the extracellular matrix (ECM) and serves as a molecular anchor to proteins involved in cellular signaling pathways regulating myofiber homeostasis (6).

Since DMD is a monogenic disease, restoring muscle function by supplying a functional copy of a dystrophin gene is a highly appealing therapeutic strategy. Several preclinical and clinical programs are in development to evaluate the efficacy and safety of systemic delivery of adeno-associated viral (AAV) vectors carrying dystrophin-based constructs to striated muscles (7). These vectors have been successfully used to express transgenes in a variety of organs, such as the liver, brain, retina, and muscles, and have shown a robust and long-term expression of transgenes with superior efficacy compared with other viral or nonviral vectors. However, AAV particles are relatively small (~20 nm) and, thus, present a limited packaging capacity to sequences of less than 5 kb (8), which poses an enormous challenge to genetic disorders with larger proteins like DMD. Dystrophin muscle isoform (Dp427) is expressed from an 11.2 kb cDNA, which far exceeds the AAV maximal packaging capacity.

Therefore, tremendous efforts have been made over the last decades to study the structural organization of dystrophin, which later led to the development of a new class of dystrophin-based gene therapies. Early studies have shown that large in-frame deletions (up to 46%) within the central rod domain result in the production of a mini-dystrophin that retains functionality and protects striated muscle from mechanical damage (9). Additional phenotypical characterizations of transgenic mice demonstrate the efficacy of these mid-size dystrophins and shed light on the modular organization of dystrophin (10–12). These studies also showed that truncated dystrophins, termed micro-dystrophins (μDys), that fit within the AAV cargo capacity are stable and functional. The administration of AAV-μDys vectors into DMD animal models resulted in significant correction of muscular dystrophy (13–16). Several μDys constructs are being evaluated in the clinic, with one drug already approved by the FDA (Elevidys). Nonetheless, an increasing number of preclinical and clinical data point to incomplete muscle recovery with various μDys constructs. This suggests the need to express larger dystrophins to fully restore the functional impairment.

Recently, we described a method for delivering and expressing large dystrophins using protein *trans*-splicing (PTS) mediated by split inteins and myotropic AAV vectors (17). PTS is a natural phenomenon originally discovered in unicellular organisms by which 2 protein halves are seamlessly fused into a functional protein (18). We adapted this posttranscriptional process to express a large midi-dystrophin (midi-Dys, ΔSR5–15) or full-length dystrophin (full-Dys) (Dp427 isoform) using, respectively, a dual or triple AAV approach (17, 19). With this method, efficient reconstitution of large dystrophin was achieved using low doses of AAVMYO1 (2–4 × 10^13^ vg/kg) in both young (mildly affected) or old (severely affected) *mdx*^4cv^ mice, which restored several functional defects to normal levels. Nonetheless, the molecular characterization of the phenotypical rescue was limited to histology assessment using common staining methods, immunolabeling of dystrophin and its glycoprotein partners, or Western blotting. Although valuable to determine the protein expression and distribution, as well as the general muscle morphology, alternative methods that give deeper insight into specific defects or protein regulation may identify biomarkers that better delineate the stages of disease progression and serve as outcome measures in clinical trials conducted using AAV-dystrophin approaches.

Here, we describe a sensitive mass spectrometry–based (MS-based) proteomics workflow that allows a holistic analysis of protein expression of wild-type (WT), saline-, or AAV-treated *mdx*^4cv^ mice. Our data revealed subtle changes in dystrophic muscles expressing different dystrophin-based constructs and led to the identification of cellular biomarkers with variable expression profiles.

## Results

### Validation of dystrophin gene therapy replacement.

**Figure 1:**
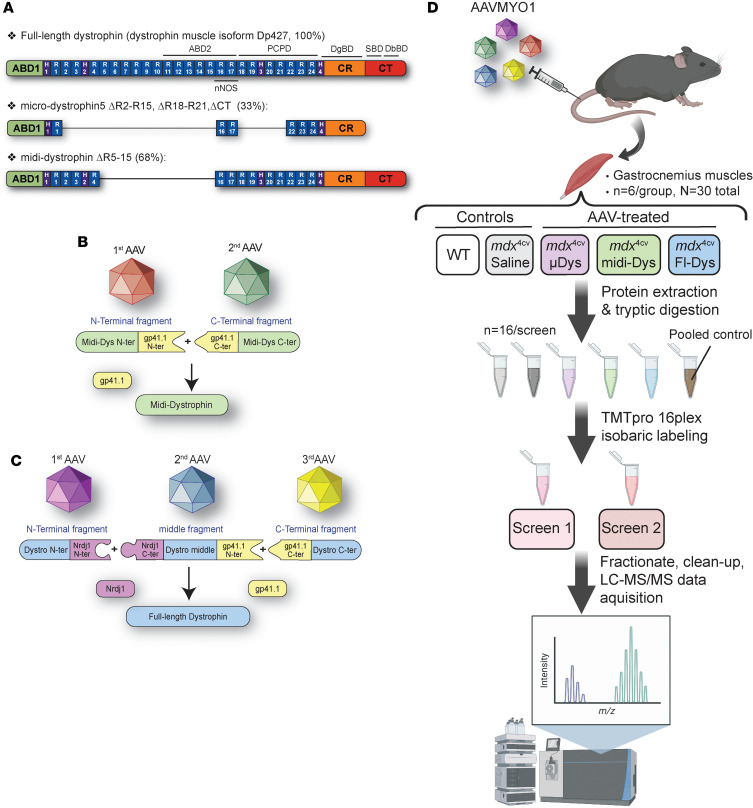
Schematic representation of dystrophin clones tested, split intein approach to express large constructs, and proteomics workflow. (**A**) Structural organization of full-length dystrophin (muscle isoform Dp427), μDys currently evaluated in clinical trials, and midi-dystrophin (ΔSR5–15). (**B**) Dual vector strategy to express a midi-dystrophin using split intein gp41.1. (**C**) Triple vector strategy to reexpress full-length dystrophin using 2 orthogonal split inteins Nrdj1 and gp41.1. (**D**) Workflow for characterization of the protein expression profile in *mdx*^4cv^ skeletal muscle. Gastrocnemius muscles were isolated from WT, saline-treated *mdx*^4cv^, or systemically injected *mdx*^4cv^ with low doses of AAVMYO1. Total proteins from 6 muscles per group were extracted and labeled with TMT isobaric tags before protein quantification using LC-MS/MS. Fl-Dys, full-length dystrophin.

We employed an isobaric labeling multiplex discovery proteomics approach to compare the skeletal muscle proteomes of healthy (WT), dystrophic (*mdx*^4cv^), and AAVMYO1-treated *mdx*^4cv^ mice with variable dystrophin constructs. Myotropic AAVMYO1 vectors were administered systemically into 8-week-old mice at low doses of 2 × 10^13^ vg/kg to express μDys5 (ΔSR2–15, Δ18–21, ΔCT) from a single vector or midi-Dys (ΔSR5–15) from dual vector, whereas triple AAVMYO1 were mixed and injected at a total dose of 4 × 10^13^ vg/kg to express full-Dys (Figure 1, A–C). Three months later, gastrocnemius muscles were collected from 6 AAV-treated mice as well as age-matched saline-treated *mdx*^4cv^ and WT mice. Protein lysates were extracted and labeled with TMT isobaric tags, and 2 proteomics screens were conducted (Figure 1D).

**Figure 2:**
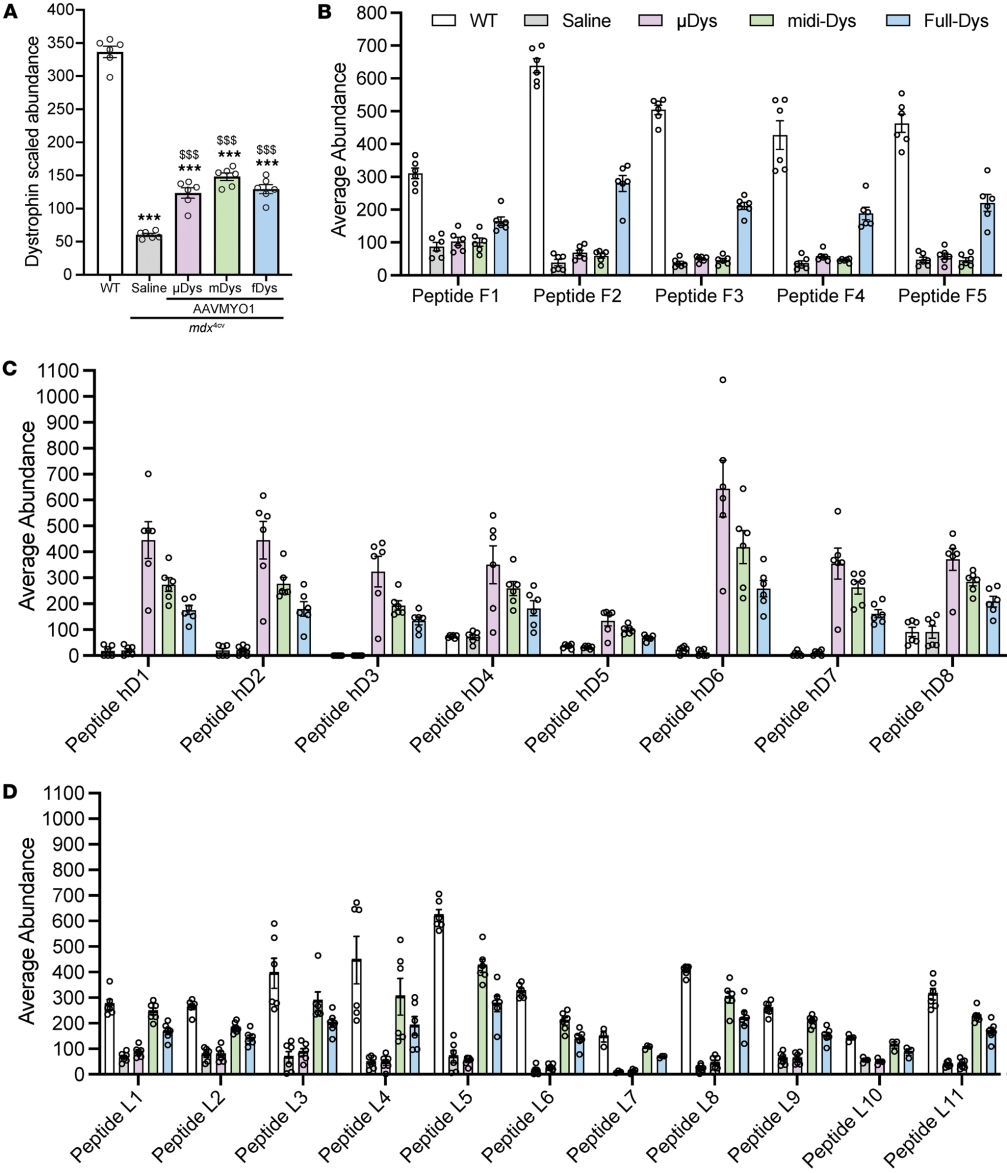
Detection of dystrophin expression and quantification of peptide-specific abundance using proteomics. Dystrophin peptide abundances were quantified using TMT proteomics in gastrocnemius muscle samples of WT, *mdx*^4cv^ treated with saline or AAVMYO1 to express μDys-*mdx*^4cv^, midiDys-*mdx*^4cv^, and full-Dys-*mdx*^4cv^. (**A**) Quantified abundance of AAV-mediated dystrophin expressed in *mdx*^4cv^ mice versus endogenous full-Dys in WT mice. (**B**) Abundance of peptides present only in full-Dys (endogenous dystrophin in WT and full-Dys construct via triple AAVMYO1 treatment). (**C**) Abundance of peptides specific to transgenic/human dystrophins (shared between μDys5, midi-Dys, and full-Dys constructs) delivered by AAVMYO1. (**D**) Abundance of peptides specific to large dystrophins (endogenous WT dystrophin, or AAV-delivered midi-Dys and full-Dys constructs). The non-zero value for dystrophin peptides in saline-treated *mdx*^4cv^ is most likely due to coisolation interference common to TMT proteomics analyses. Bar graphs depict mean ± SEM of *n* = 6 mice/group, except peptide L7 and L10, which were *n* =3. Comparisons between groups were made using 1-way ANOVA with Tukey’s multiple-comparison test. ***P < 0.001 versus WT; ^$$$^*P* < 0.001 versus *mdx*^4cv^ saline. μDys: micro-dystrophin, mDys and midi-Dys: midi-dystrophin, fDys and full-Dys: full-length dystrophin.

To verify dystrophin expression in each experimental group, construct-specific dystrophin peptide abundances were assessed (Table 1). Transgenic dystrophin constructs were detected in the samples from *mdx*^4cv^ mice treated with single, dual, or triple AAVs, but at lower abundance versus endogenous dystrophin in WT muscles (Figure 2A). As expected, the average abundance of peptide sequences specific to full-Dys was found elevated exclusively in WT or triple-AAV groups (Figure 2B). Similarly, peptide sequences specific to transgenic/human dystrophins (μDys5, intein-generated midi-Dys, or full-Dys) were elevated across all AAV treatment groups (Figure 2C). Using different peptides, μDys and midi-Dys were detected at comparable levels, whereas average full-Dys abundance was slightly lower. Finally, by searching peptides specific to large dystrophin (i.e., excluding μDys), we confirmed the exclusive expression of large dystrophins in WT or *mdx*^4cv^ mice treated with dual or triple AAV vectors (Figure 2D). Overall, the abundance of the dystrophins was peptide dependent, with a variable sensitivity observed from one peptide to another.

Together, these data highlight the specificity of this approach to detecting and quantifying endogenous or ectopic dystrophin proteins using specific sequences in healthy or dystrophic muscles after AAV treatment with different gene replacement approaches.

### Muscle histology improvement after dystrophin expression.

**Figure 3:**
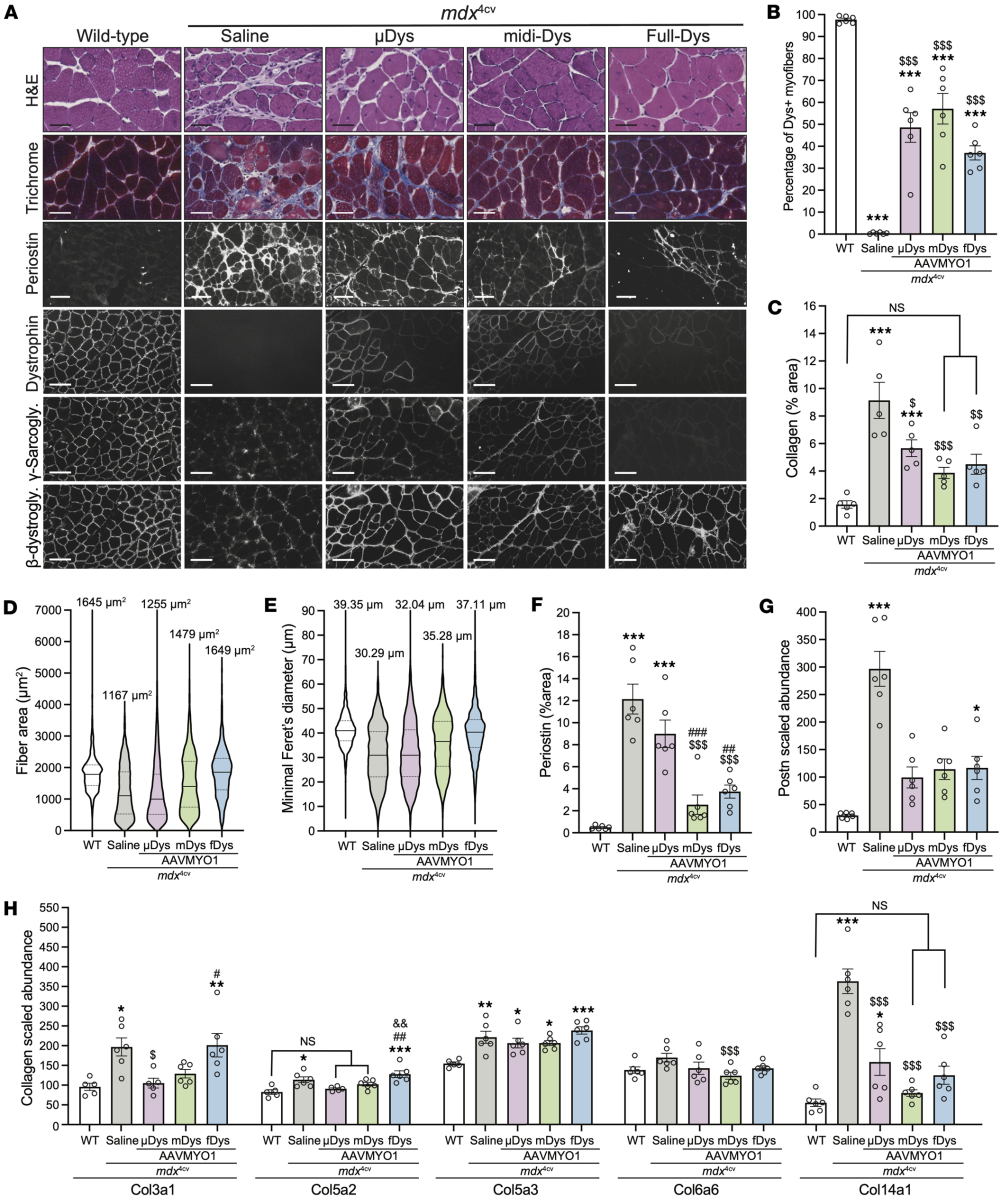
Histology analysis of gastrocnemius muscle cross sections showing improvements with dystrophin constructs. (**A**) Representative images of gastrocnemius muscle cross sections stained with H&E or trichrome (top rows, scale bars: 50 μm), or immunolabeled with antibodies specific for periostin (scale bars: 50 μm) or dystrophin-glycoprotein elements (lower panel, scale bars: 100 μm). These images were acquired in RGB colors but inverted to black and white for better visualization. The original panel is presented in Supplemental Figures 2 and 3. (**B**) Percentage of dystrophin-positive fibers; 600–1000 myofibers were counted per sample, with *n* = 6 analyzed per group. (**C**) The collagen area of the gastrocnemius muscle was measured using trichrome-stained cross sections. *n* = 5 samples per group. (**D**) Gastrocnemius myofiber area and (**E**) minimal Feret’s diameter. More than 700 myofibers per sample from *n* = 6 per group were analyzed. The average values are shown on top of the violin bars. The solid line represents the median, while the dashed lines show the quartiles. (**F**) Periostin area measured from cross-section muscle sections immunolabeled with specific antibodies against periostin. *n* = 6 samples per group. (**G**) Periostin abundance level detected from proteomics analysis of gastrocnemius muscles. (**H**) Abundance levels of different collagens were measured using the proteomics method from gastrocnemius samples. NS, not significant. *P < 0.05, **P < 0.01, ***P < 0.001 versus WT; ^$^*P* < 0.05, ^$$^*P* < 0.01, ^$$$^*P* < 0.001 versus saline group; ^#^*P* < 0.05, ^##^*P* < 0.01, ^###^*P* < 0.001 versus μDys group; ^&&^*P* < 0.01 versus midi-Dys group using 1-way ANOVA followed by Tukey’s post hoc test. Dys^+^: dystrophin-positive. H&E, hematoxylin and eosin; μDys, micro-dystrophin; mDys and midi-Dys, midi-dystrophin; fDys and full-Dys, full-length dystrophin.

To evaluate the muscle histology and compare the therapeutic benefits of each gene replacement modality, serial cross sections of gastrocnemius were stained with hematoxylin and eosin (H&E), trichrome, or immunolabeled using specific antibodies raised against elements of the DGC or periostin. In the group treated with AAVs, dystrophin expression was detected in 40%–60% of myofibers, whereas a few revertant fibers, not exceeding 1%, were found in the saline group (Figure 3, A and B). As gastrocnemius muscles are predominantly composed of fast-twitch myofiber type II, more than 86% of dystrophin-positive fibers were either type IIa, IIb, or IIx (Supplemental Figure 1; supplemental material available online with this article; https://doi.org/10.1172/jci.insight.197759DS1). Muscles from animals treated with saline presented typical dystrophic muscle histology with small fibers and fibrotic and infiltrated muscle tissue compared with WT muscles (Figure 3, A, and C–E). In contrast, muscle from *mdx*^4cv^ mice treated with AAVs showed improved histology with a substantial increase in myofiber area and diameter, with the highest values observed with large dystrophins (i.e., midi- and full-Dys). A marked reduction in collagen content was also found in groups treated with AAVs (Figure 3, A and C). Similarly, immunolabeling of periostin showed an increased area in saline-treated dystrophic muscles, confirming the expansion of the ECM, which AAV-dystrophin treatment prevented (Figure 3F). Interestingly, while proteomics data confirmed the upregulation of periostin (Figure 3G), variable abundance of collagen isoforms was observed. For instance, collagen isoform I (α1 and α2), IV (α1 and α2), VI (α1, α2, and α3), and XII abundance was unchanged among groups, whereas collagen type III (α1), V (α2 and α3), VI (α6), and XIV (α1 chain) abundance was elevated in *mdx*^4cv^ muscle and restored by AAV treatment (Figure 3H and Supplemental Figure 2).

### Characterization of molecular changes using proteomics.

**Figure 4:**
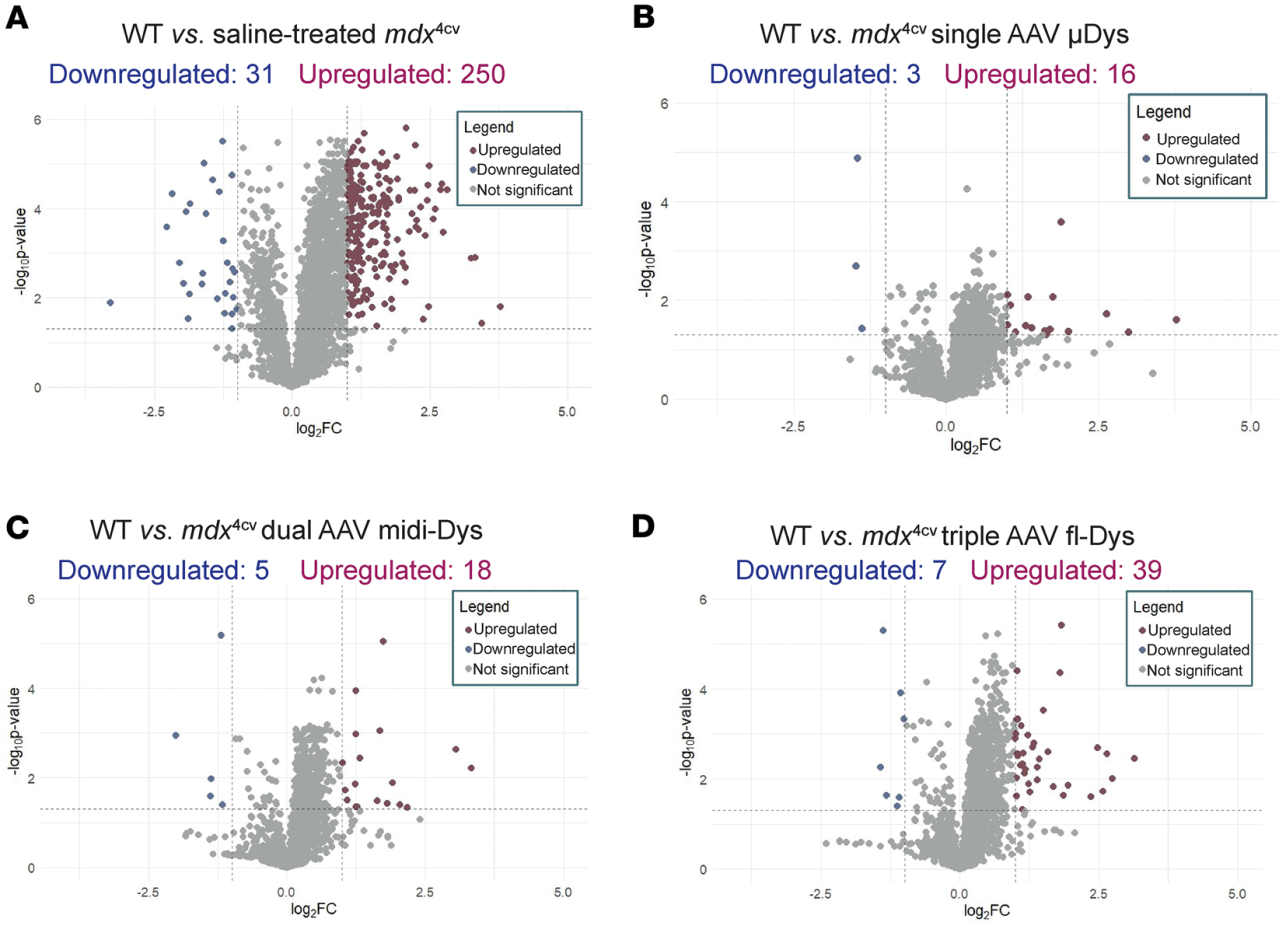
Comparison of protein expression profiles between experimental groups. Protein expression profiles in gastrocnemius muscle were compared between WT mice and (**A**) saline-*mdx*^4cv^, (**B**) *mdx*^4cv^ injected with single AAVMYO1 μDys, (**C**) *mdx*^4cv^ injected with dual AAVMYO1 to express midi-dystrophin, or (**D**) *mdx*^4cv^ injected with triple AAVMYO1 vector to express full-length dystrophin. A 2-tailed, unpaired Student’s *t* test was used to calculate *P* values for pairwise fold changes, and the Benjamini-Hochberg method was used to control the false discovery rate (FDR). Corrected *P* values were log-transformed and plotted against log-transformed fold change values to obtain volcano plots, and a minimum corrected *P*-value cutoff of 0.05 and minimum relative fold change cutoff of ±1 were applied to identify differentially expressed proteins (DEPs) in pairwise comparisons. Data were collected from a sample size of *n* = 6 per group.

Next, we investigated general trends in protein expression profiles between WT, *mdx*^4cv^, and AAV treatment groups. Dystrophin-deficient *mdx*^4cv^ gastrocnemius muscle displayed a large number of differentially expressed proteins (DEPs) compared with WT muscle, including 250 upregulated proteins and 31 downregulated proteins (Figure 4A). The top upregulated and downregulated pathways in *mdx*^4cv^ muscle have been previously reported in *mdx* mice, demonstrating defects in, for example, cytoskeletal structure and sarcolemmal integrity (6, 20), ECM organization (21–23), and fatty acid metabolism (24, 25).

In contrast, few proteins displayed significantly elevated or depleted levels in single, dual, or triple AAV–treated *mdx*^4cv^ gastrocnemius muscle compared with WT muscle (Figure 4, B–D). A total of 16 upregulated and 3 downregulated proteins were identified between μDys5-*mdx*^4cv^ and WT mice (Figure 4B). Eighteen upregulated proteins and 5 downregulated proteins were observed in midi-Dys-*mdx*^4cv^ muscle compared with WT (Figure 4C), while 39 upregulated and 7 downregulated proteins were found between full-Dys-*mdx*^4cv^ and WT mice (Figure 4D).

**Figure 5:**
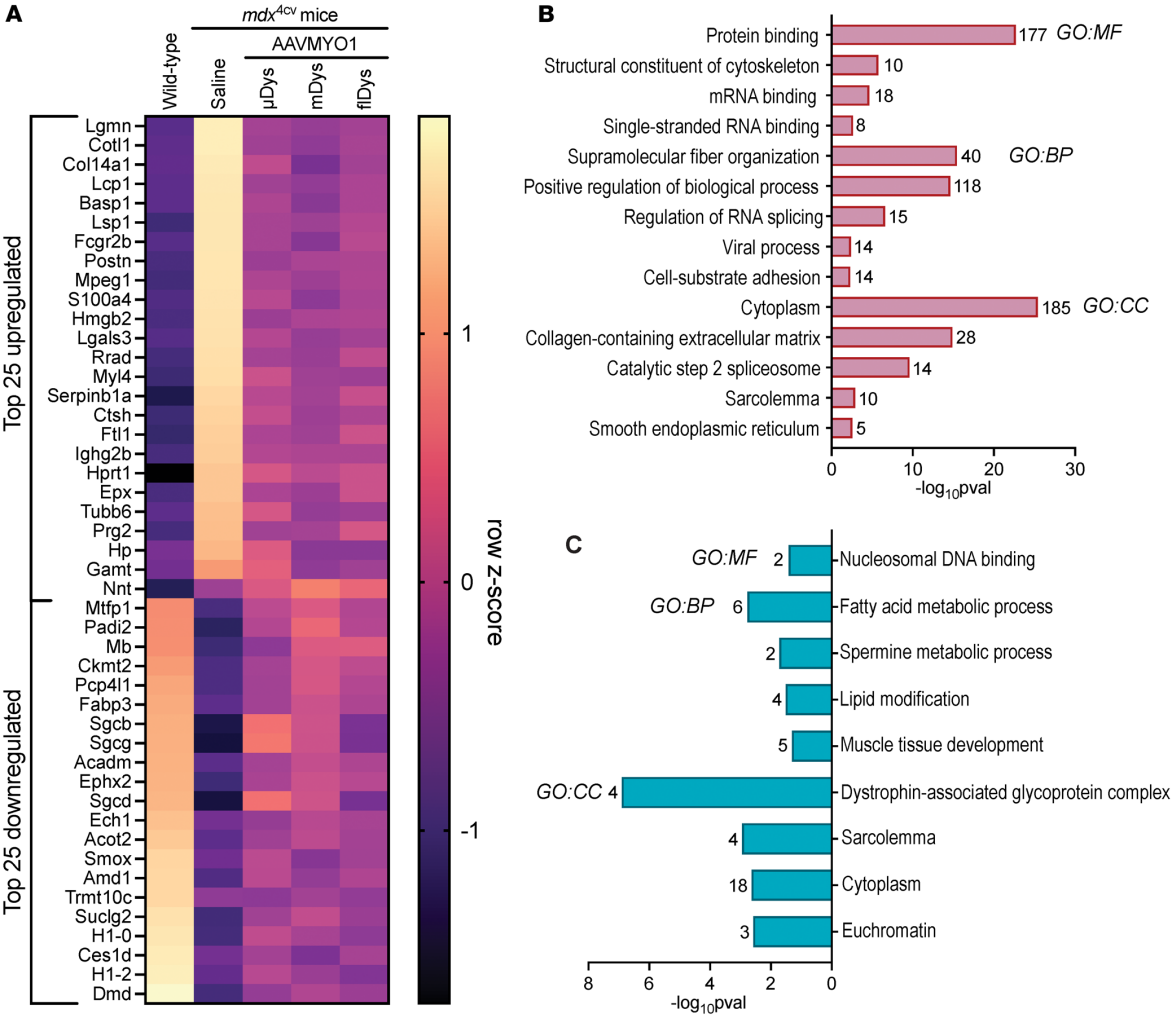
Analysis of protein expression profile demonstrates proteomic rescue by dystrophin constructs. (**A**) Heatmap depicting the top upregulated and downregulated proteins between WT and *mdx*^4cv^ muscle. Gene ontology (GO) enrichment analysis was performed using GOrilla and g:Profiler to determine the molecular function (MF), biological process (BP), and cellular compartment (CC) enrichment of significantly (**B**) upregulated and (**C**) downregulated proteins in *mdx*^4cv^ gastrocnemius muscle compared with WT muscle.

Further analysis of top upregulated and downregulated DEPs in *mdx*^4cv^ compared with WT gastrocnemius revealed that several cellular processes were dysregulated (Figure 5). For instance, upregulated DEPs in *mdx*^4cv^ muscle were enriched for molecular functions and biological processes, including protein and mRNA binding, cytoskeletal structure, supramolecular fiber organization, and regulation of RNA splicing, with cellular compartment enrichment for cytoplasmic, collagen-containing ECM, spliceosome, sarcolemmal, and endoplasmic reticulum proteins (Figure 5, A and B). Downregulated DEPs in *mdx*^4cv^ muscles, however, were enriched for molecular functions including nucleosomal DNA binding, fatty acid metabolic processes, and muscle tissue development, with the cellular compartment enrichment for DGC, sarcolemmal, cytoplasmic, and euchromatin-enriched proteins (Figure 5, A and C).

**Figure 6:**
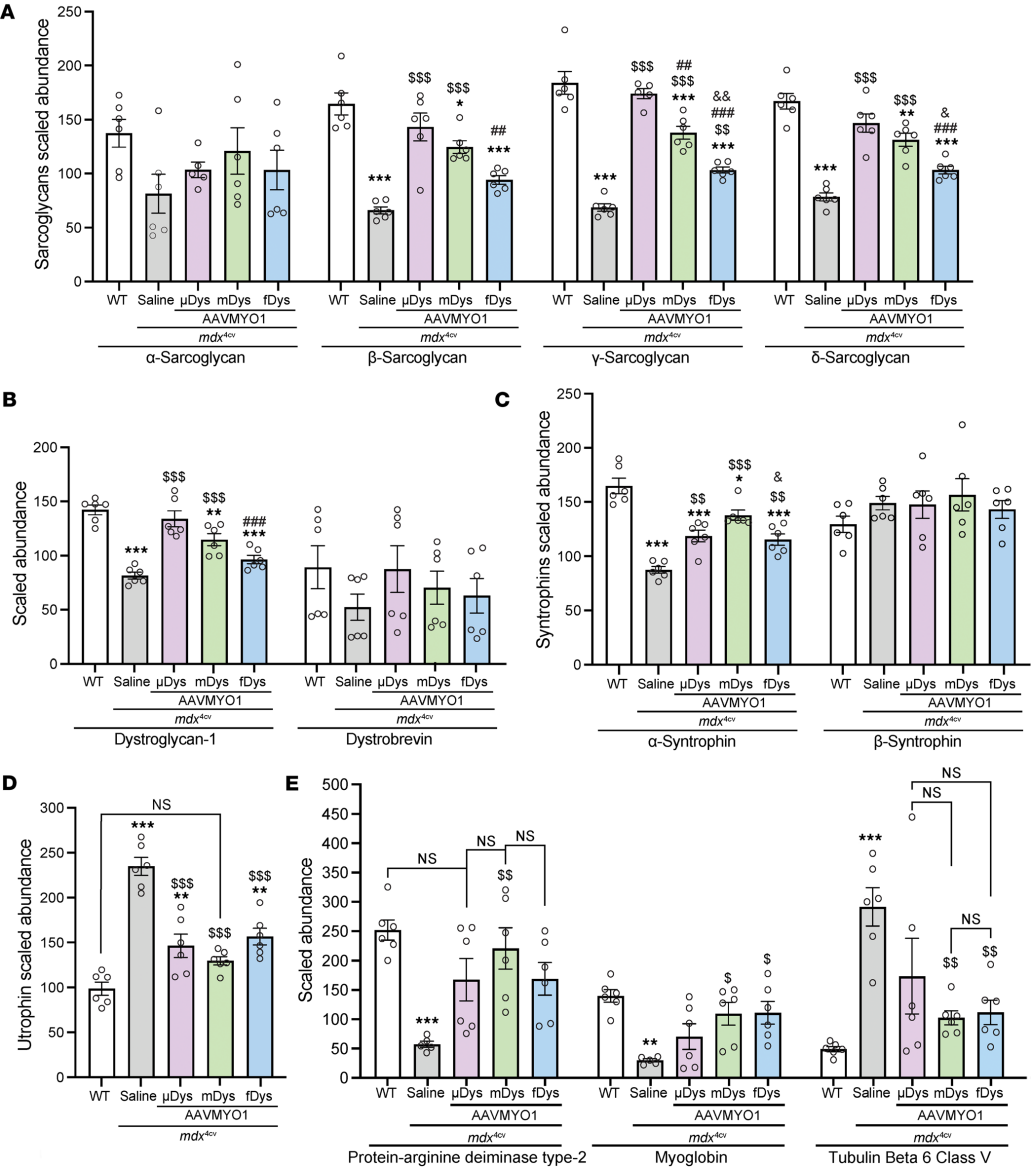
Dystrophin delivery alleviates DGC protein defects in *mdx*^4cv^ mice. Relative abundance of (**A**) sarcoglycans, (**B**) dystroglycan, dystrobrevin, (**C**) syntrophins, (**D**) utrophin, and (**E**) protein-arginine deiminase type-2, myoglobin, and tubulin β6 class V measured by the proteomics method. Bar graphs depict mean ± SEM from *n* = 5–6 mice/group. Comparisons between groups were made using 1-way ANOVA with Tukey’s multiple-comparisons test. NS, not significant. *P < 0.05, **P < 0.01, ***P < 0.001 versus WT; ^$^*P* < 0.05, ^$$^*P* < 0.01, ^$$$^*P* < 0.001 versus saline group; ^##^*P* < 0.01, ^###^*P* < 0.001 versus μDys group; ^&^*P* < 0.05, ^&&^*P* < 0.01 versus midi-Dys. μDys, micro-dystrophin; mDys, midi-dystrophin; fDys, full-length dystrophin.

Importantly, several of these defects were partially restored with the dystrophin replacement using AAVMYO1 vectors at variable levels (Figures 5 and 6). For example, treatment with AAV-μDys5 restored the abundance of DGC proteins, including sarcoglycans (β, γ, and δ) and dystroglycans (Figure 3A, Figure 6, A and B, and Supplemental Figures 3 and 4), while the dual AAV-midi-Dys and triple AAV-full-Dys treatments resulted in similar patterns of proteomic restoration compared to *mdx*^4cv^ muscle but were slightly less effective in restoring sarcoglycan and dystroglycan levels (Figure 3A and Figure 6, A and B). In contrast, levels of α-syntrophin and utrophin were normalized with dual AAV-midi-Dys but remain slightly affected with μDys or triple AAV-full-Dys treatments (Figure 6, C and D, and Supplemental Figure 4), although utrophin levels were variable when assessed by Western blot (Supplemental Figure 4).

Similarly, several proteins with elevated abundance in WT muscle displayed reduced abundance in saline-treated *mdx*^4cv^, whereas dystrophin construct expression mediated by AAV partially or fully restored their cellular enrichment, including protein-arginine deiminase type-2 and myoglobin (Figure 6E), as previously shown for myoglobin (26). In contrast, tubulin β6 class V, whose abundance was higher in the saline group, consistent with a previous study (27), was greatly reduced in AAV-treated groups (Figure 6E).

In summary, these data confirm the depletion of the DGC in *mdx*^4cv^ muscle and corroborate other known disease sequelae in dystrophin-deficient muscle, including increased fibrosis and collagen deposition in the ECM, whereas μDys and intein-generated midi-Dys and full-Dys, respectively, restored 262, 258, and 235 out of 281 dysregulated proteins, which greatly improved the underlying cellular defects in *mdx*^4cv^ mice.

### Dystrophin replacement partially restores biomarkers involved in membrane repair and myogenesis.

**Figure 7:**
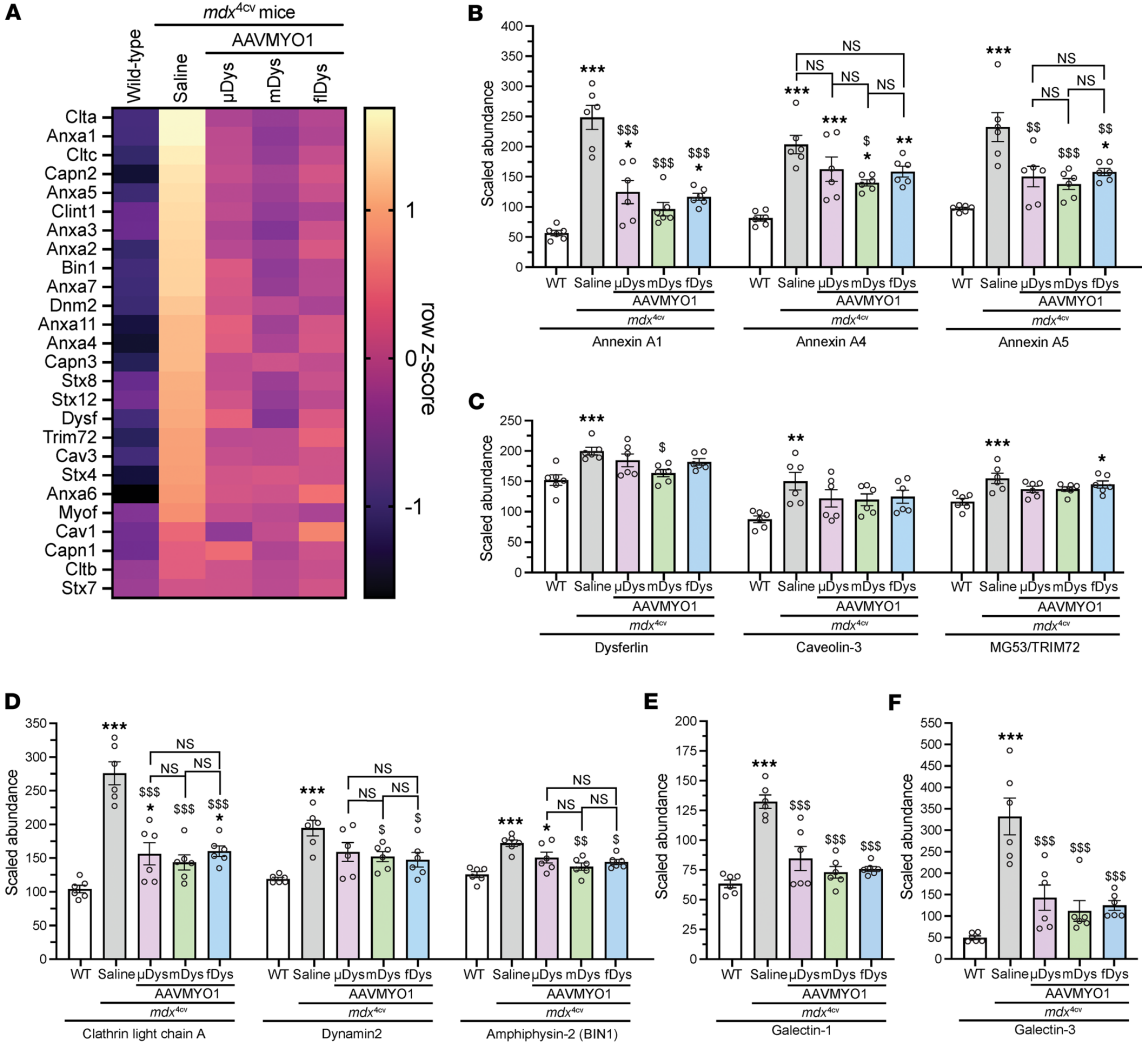
Amelioration of altered membrane repair and myogenesis pathway markers in *mdx*^4cv^ muscle mediated by AAV-dystrophin constructs. (**A**) Heatmap showing elevated expression of various proteins implicated in membrane trafficking and repair in *mdx*^4cv^ gastrocnemius muscle and partial restoration with μDys5, midi-dystrophin, or full-length dystrophin delivered by AAV vectors. (**B**) Annexin (A1, A4, and A5) abundance in WT, dystrophic, or AAV-treated muscles. (**C**) Abundance of proteins involved in muscle repair. (**D**) Expression of key proteins involved in membrane trafficking and remodeling. (**E**) Galectin-1 and (**F**) galectin-3 abundance in *mdx*^4cv^ and WT muscles. Bar graphs represent mean ± SEM of *n* = 6 mice/group. NS, not significant. *P < 0.05, **P < 0.01, ***P < 0.001 versus WT; ^$^*P* < 0.05, ^$$^*P* < 0.01, ^$$$^*P* < 0.001 versus saline group using 1-way ANOVA followed by Tukey’s post hoc test. μDys, micro-dystrophin; mDys, midi-dystrophin; fDys, full-length dystrophin.

Severe sarcolemmal fragility and susceptibility to cycles of damage and muscle regeneration represent a hallmark of DMD pathology due to the absence of dystrophin as a structural membrane protein. Disease-specific proteomic changes in *mdx* skeletal muscle include changes in cytoskeletal, structural, and membrane repair proteins (28). Based on our data demonstrating that more than 85% of *mdx*^4cv^ proteomic changes exhibit an intermediate or near-complete level of rescue by various-length AAV-Dys treatment, we investigated the impact of μDys5, midi-Dys, and full-Dys expression on membrane trafficking and repair proteins in *mdx*^4cv^ gastrocnemius muscle. A general trend of pathway elevation was observed in *mdx*^4cv^ muscle, with partial restoration across all AAV-Dys treatment groups (Figure 7A). Following this pattern, annexin A1 and annexin A5 levels were increased in *mdx*^4cv^ muscle compared with WT and partially restored by AAV-Dys treatment (Figure 7B). We also observed elevated annexin A4 levels in *mdx*^4cv^ muscle, but only AAV-midi-Dys treatment significantly reduced annexin A4 to an intermediate level between WT and dystrophin-deficient muscle (Figure 7B). Dysferlin also displayed elevated levels in saline-*mdx*^4cv^ muscles. While an intermediate restoration was detected in the AAV-treated groups, only dual midi-Dys treatment significantly reduced dysferlin levels compared to saline-*mdx*^4cv^ (Figure 7C and Supplemental Figure 4). Likewise, elevated levels of caveolin-3 and MG53/TRIM72 were found in control *mdx*^4cv^ muscle that were significantly but modestly reduced by AAV treatment (Figure 7C).

Furthermore, we analyzed the expression level of proteins implicated in membrane remodeling, trafficking, and cytoskeleton dynamics, such as clathrin light chain A, dynamin-2, and amphiphysin-2 (BIN1). These proteins were enriched in saline-*mdx*^4cv^ muscles, with 2- to 3-fold higher levels compared with WT muscles (Figure 7D). However, variable effects were found with the different dystrophin constructs. For instance, partial restoration was observed with the single AAV-μDys treatment, whereas near-complete normalization of these proteins was obtained with dual or triple AAV approaches (Figure 7D). Conversely, all dystrophin constructs restored the level of galectin-1 to WT levels and significantly reduced galectin-3, which were found to be 3- and 5-fold higher, respectively, in saline-treated dystrophic muscles (Figure 7, E and F).

These observations highlight the impairment of several key proteins involved in different pathways, including myogenesis, membrane repair, and remodeling in dystrophin-deficient myofibers, which were rescued to variable extents by dystrophin replacement strategies using single, dual, or triple AAVMYO1.

### Incomplete corrections with dystrophin gene therapy.

**Figure 8:**
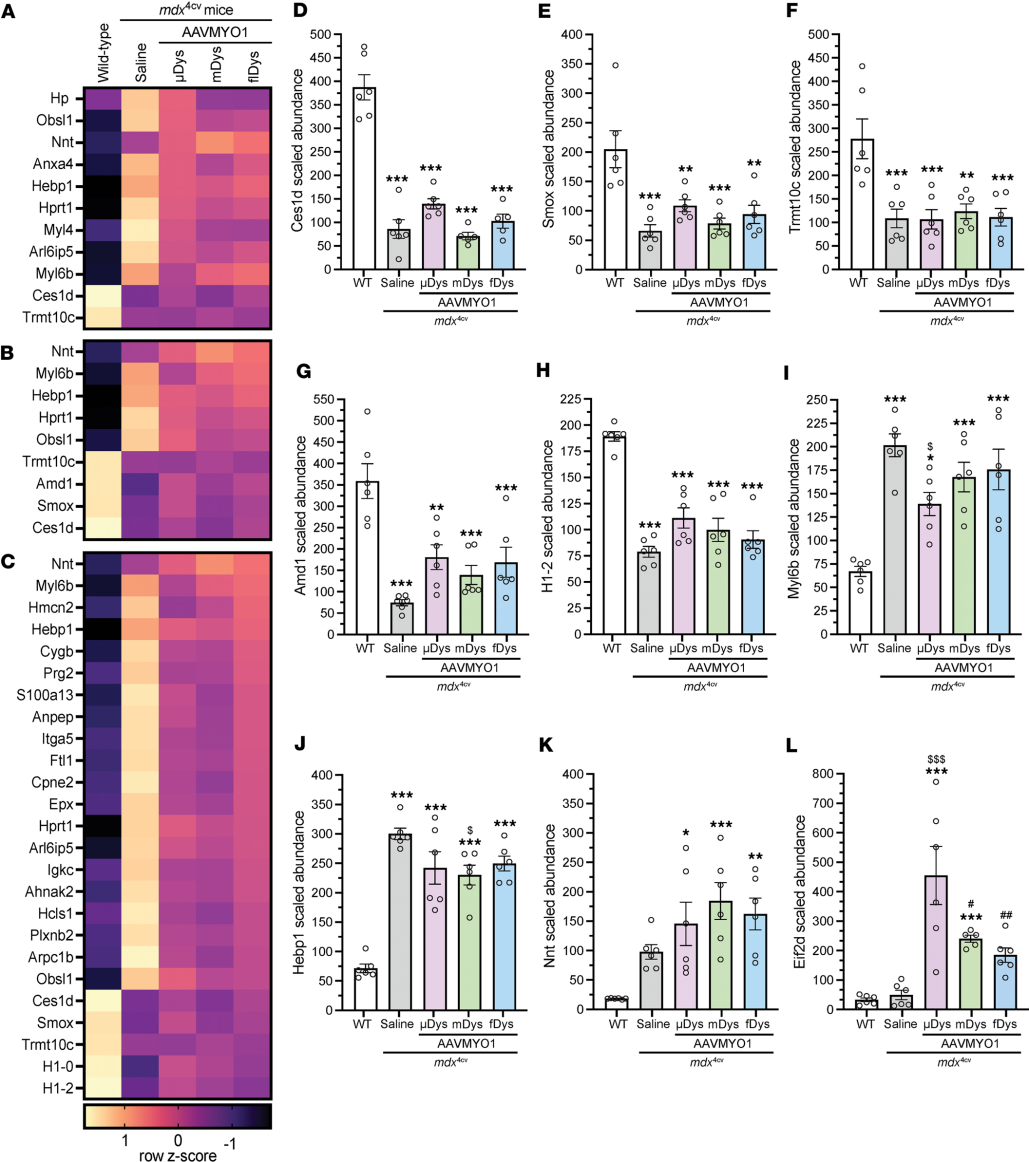
Proteins with unrestored expression in *mdx*^4cv^ mice treated with various dystrophin constructs. Heatmaps displaying proteins that did not display significant restoration to WT levels in (**A**) μDys5-*mdx*^4cv^, (**B**) midi-Dys-*mdx*^4cv^, or (**C**) full-Dys-*mdx*^4cv^ gastrocnemius muscles. Exemplary proteins with unrestored levels in AAV-Dys construct groups include (**D**) carboxylesterase 1D (Ces1d), (**E**) spermine oxidase (Smox), (**F**) tRNA methyltransferase 10 homolog C (Trmt10c), (**G**) adenosylmethionine decarboxylase (Amd1), (**H**) histone H1.2 (H1-2), (**I**) myosin light chain 6B (Myl6b), (**J**) heme-binding protein (Hebp1), (**K**) nicotinamide nucleotide transhydrogenase (Nnt), and (**L**) eukaryotic translation initiation factor 2D (Eif2d). Bar graphs depict mean ± SEM from *n* = 5–6 mice/group. Comparisons between groups were made using 1-way ANOVA with Tukey’s multiple-comparison test. *P < 0.05, **P < 0.01, ***P < 0.001 versus WT; ^$^*P* < 0.05, ^$$$^*P* < 0.001 versus saline; ^#^*P* < 0.05, ^##^*P* < 0.01 versus μDys group. μDys, micro-dystrophin; mDys, midi-dystrophin; fDys, full-length dystrophin.

Based on the observation that *mdx*^4cv^ gastrocnemius muscles treated with single, dual, or triple AAV-Dys constructs retain some proteomic features that are distinct from healthy WT muscle (Figure 4), we sought to identify whether AAV–split-intein-Dys treatment results in unique, potentially pathological changes in protein expression and whether the unrestored DEPs in AAV-treated *mdx*^4cv^ muscle are relevant to DMD disease processes. We filtered our dataset for proteins that met the following 2 criteria: (a) significantly altered in AAV-treated *mdx*^4cv^ muscle compared with WT muscle, and (b) not significantly altered between AAV-treated and untreated *mdx*^4cv^ groups. After filtering, we obtained short lists of unrestored DEPs in μDys5-*mdx*^4cv^, midi-Dys-*mdx*^4cv^, and full-Dys-*mdx*^4cv^ gastrocnemius muscle (Figure 8, A–C). Several proteins demonstrated depleted abundance in *mdx*^4cv^ muscle that was not restored by the different dystrophin constructs, including carboxylesterase 1D (gene name *Ces1d*; Figure 8D), spermine oxidase (gene name *Smox*; Figure 8E), tRNA methyltransferase 10 homolog C (gene name *Trmt10c*; Figure 8F), adenosylmethionine decarboxylase (gene name *Amd1*; Figure 8G), and histone H1.2 (gene name *H1-2*; Figure 8H). Levels of several upregulated proteins in *mdx*^4cv^ muscle were not ameliorated or were only partially ameliorated by AAV-dystrophin treatments, including myosin light chain 6B (gene name *Myl6b*; Figure 8I) and heme-binding protein 1 (gene name *Hebp1*; Figure 8J). Importantly, the introduction of split-intein dystrophin constructs did not induce unique or deleterious proteomic changes in the *mdx*^4cv^ gastrocnemius muscles. A singular protein, nicotinamide nucleotide transhydrogenase (NNT; gene name *Nnt*), demonstrated expression changes in *mdx*^4cv^ muscle that were more pronounced with AAV-dystrophin treatment; however, NNT expression levels did not display a statistically significant difference between treated and untreated *mdx*^4cv^ muscle (Figure 8K). Only 2 of the proteins identified as dysregulated in naive or AAV-treated *mdx*^4cv^ muscle, myosin light chain 4 (gene name *Myl4*) and hypoxanthine-guanine phosphoribosyltransferase (gene name *Hprt1*), were referenced in previous studies involving *mdx* mice (29–32). Notably, a singular protein, eukaryotic translation initiation factor 2D (gene name *Eif2d*), was identified as uniquely altered by AAV treatment (Figure 8L), suggesting a minimal biological impact of injection with the AAV constructs themselves.

## Discussion

Genetic mutations in the *DMD* gene have been associated with the development of dystrophinopathies, a group of fatal diseases characterized by progressive degeneration of striated muscles. While the primary cause is the lack of functional dystrophin, leading to fragility of the sarcolemma membrane and high susceptibility to damage from muscle contraction, additional cellular defects are being revealed through studies involving patient-derived biological material or dystrophin-deficient cellular and animal models (33). A plethora of therapeutic strategies have emerged aiming to deliver or restore the expression of dystrophin or treat downstream disease sequelae by modulating several signaling pathways (34). However, measuring the effectiveness of these therapies was limited to the quantitation of dystrophin protein, the characterization of the general muscle histology, or measuring the mechanical properties of skeletal muscle. Here, we utilized a proteomics method to delineate a global protein expression profile in healthy or dystrophin-deficient murine muscles. Moreover, we used this method to validate the therapeutic outcomes of 3 different dystrophin replacement strategies in *mdx*^4cv^ mice and compiled a list of unrestored defects that might be used as biomarkers for future studies.

Our dataset confirms the depletion of DGC proteins and demonstrates an overall pattern of elevated expression for membrane trafficking and repair pathway proteins in *mdx*^4cv^ muscle, with partial restoration of some proteins by the different dystrophin constructs expressed via AAV delivery. Lower levels of DGC proteins were previously described in dystrophin-null muscles as a direct consequence of the absence of dystrophin (35, 36), whereas the upregulation of MG53/TRIM72, dysferlin, caveolin-3, utrophin, and members of the annexin family may reflect an adaptive response to increased sarcolemmal membrane fragility and rupture. The function of annexin A4 in sarcolemmal repair has not been clearly defined. However, overexpression of other annexin family members has been observed in DMD muscle as a response to increased membrane fragility and activation of membrane repair processes (37, 38). Interestingly, genetic mutations affecting the expression of dysferlin and caveolin-3 have been associated with the development of muscular dystrophies (39–42). In addition, several reports have shown the molecular interaction of MG53/TRIM72, dysferlin, and caveolin-3 in muscles and suggested their modulation as a potential therapeutic target in various muscular disorders (43–47).

Similarly, other proteins with essential roles in membrane trafficking and remodeling, including clathrin light chain 1, dynamin-2, and amphiphysin-2 (BIN1), were found expressed at high levels in dystrophin-null *mdx*^4cv^ muscles. These proteins were also linked to the pathogenesis of different congenital myopathies (48, 49). In the last decade, dynamin-2 and BIN1 have been extensively investigated as genetic modifiers in different muscular disorders (50–54), but their role in the pathogenesis of DMD has yet to be characterized. For instance, BIN1 and dynamin-2, as well as dysferlin and caveolin-3, are involved in transverse tubule (T-tubule) formation (55). These invaginations of the sarcoplasmic membrane associate with 2 sarcoplasmic reticula to form the triads, which are key regulators in excitation-contraction coupling. Early studies suggested the presence of dystrophin in the T-tubules (56, 57), while another study indicated structural and functional defects in the sarcoplasmic reticulum in dystrophin-deficient muscles, contributing to calcium homeostasis defects (58). Although the expression of dystrophin constructs with variable lengths using AAV vectors leads to changes in the expression profiles of the various proteins involved in membrane repair, trafficking, and remodeling, additional studies confirming the restoration of these cellular processes are needed.

Our data confirm the upregulation of galectin-1 and galectin-3. Elevated levels of these proteins have been reported previously in cellular and animal models of DMD, as well as in patient-derived muscle samples (59–61). Recent work has linked galectin-3 to lysosomal damage in 2 mouse models of muscular dystrophy (61). In particular, AAV-mediated γ-sarcoglycan gene replacement normalized galectin-3 expression in the *Sgcg^–/–^* mouse model of limb-girdle muscular dystrophy R5 (LGMDR5, γ-sarcoglycanopathy). In contrast, μDys supplementation in *mdx*^4cv^ mice resulted in limited rescue of lysosomal defects, which were hypothesized to arise from elevated galectin-3 levels (61). Our proteomic data demonstrate restoration of galectin-3 across all dystrophin constructs tested, including μDys. Differences in μDys sequence, expression cassette, AAV capsid (AAV9 versus the myotropic vector AAVMYO), and vector dose may underlie the divergent outcomes observed in *mdx*^4cv^ mice between the 2 studies. Nevertheless, our dataset lacks histopathological characterization of lysosomal damage–mediated defects. Further studies are warranted to elucidate the impact of these defects in skeletal muscle and to assess the therapeutic potential of different dystrophin constructs.

Additionally, using our proteomics approach, we correlated the increase in fibrosis found on muscle sections stained with trichrome to the upregulation of collagen XIVα1, but not other collagen isoforms. Collagen XIVα1 plays a crucial role in the regulation of ECM organization and tissue integrity across various organs and has been linked to fibrotic disease as well as cardiovascular conditions (62). Nonetheless, most studies agree on the primary implication of collagen I (α1 and α2 chains) and collagen III in the development of fibrosis in skeletal muscles (63–65). The time point chosen in this study (i.e., 5 months of age) is too premature to draw robust conclusions about the expression profile of the different collagen forms and their contributions to the mild and early-stage fibrosis found in the muscle sections. Moreover, our data confirmed the upregulation of periostin, which was previously identified as a profibrotic marker in *mdx* mice and other mouse models of muscular dystrophies (66, 67).

Furthermore, AAV-mediated delivery and expression of dystrophin constructs did not restore the expression profile of various proteins to WT levels. This could be explained by either the mosaic expression of dystrophin in only half of myofibers or the disease status and the age when AAVs were administered (8 weeks old and analysis 3 months after AAV infusion). At this age, *mdx* muscles may have already accumulated cellular, histological, and functional defects due to the absence of dystrophin during muscle development in the embryonic stage, as well as the postnatal phase (68, 69).

It is noteworthy that clinical trials of AAV-μDys also display mosaic expression of μDys and invariably enroll patients who have already begun developing dystrophic pathophysiology. At this stage, in both mice and patients, skeletal muscles have undergone many cycles of degeneration and regeneration crisis, and many, if not all, myofibers have been replaced (70). Previous studies have shown that the downregulation of genes encoding adenosylmethionine decarboxylase (*Amd1*) and spermine oxidase (*Smox*) worsens the myopathy in the tibialis anterior muscle of mice with LAMA2-deficient congenital muscular dystrophy (71). More recent evidence suggests that dysregulated polyamine metabolism also contributes to muscle fiber defects in the context of amyotrophic lateral sclerosis (72). Increased urinary levels of spermine metabolites have been observed in patients with DMD for decades (73). However, there is not enough evidence available to support the central role of altered polyamine homeostasis in promoting skeletal muscle pathology in patients with DMD. The consequences of unrestored polyamine metabolic enzyme levels in *mdx*^4cv^ gastrocnemius muscle after AAV-dystrophin therapy are, therefore, unknown but unlikely to fully explain residual functional deficits in treated muscle.

In conclusion, this study describes the use of a proteomics approach to study the global protein expression in healthy or dystrophic skeletal muscles. This method can be implemented to validate therapeutic strategies in preclinical and clinical studies and monitor the effectiveness of treatments for muscular disease.

## Methods

### Sex as a biological variable.

DMD is an X-linked disease affecting mainly boys. Therefore, only male *mdx* mice were used in this study. *mdx*^4cv^ female and male mice were used for breeding and generating mouse cohorts.

### Animals.

Mice were randomized into experimental groups based on availability. They were assigned a serial identification number to conduct a blinded study. These numbers were used throughout the study, and the treatment history of each mouse was determined after completing the data collection.

### AAV production.

μDys5 (ΔSR2–15, ΔSR18–21, ΔCT), split gp41.1/midi-Dys (ΔSR5–15) N- or C-terminal constructs, or split dystrophin with split Nrdj1 and split gp41.1 combination were inserted in pAAV containing the muscle-specific M-creatine kinase (CK) 8e expression cassette (CK8e, gift from Stephen D. Hauschka, University of Washington) and a synthetic polyA flanked by 2 inverted terminal repeats (17, 19). These constructs were packaged in the myotropic AAVMYO1 (gift from Dirk Grimm, University of Heidelberg) vectors using the conventional triple plasmid transfection of HEK293 cells as previously described (74).

### AAV administration.

Eight-week-old *mdx*^4cv^ males were anesthetized using isoflurane (Piramal Critical Care) before systemic administration of a low dose of AAVMYO1 into the tail vein (μDys: 2 × 10^13^ vg/kg, midi-Dys: 1 × 10^13^ vg/kg of each vector, full-Dys: 1.33 × 10^13^ vg/kg of each vector). As a control, a subgroup was injected with sterile saline. Once AAV or saline solutions were successfully administered, mice were kept in a warm cage and monitored for 1 hour.

### Muscle histology analysis.

Gastrocnemius muscles were isolated from 5-month-old WT or *mdx*^4cv^ mice and flash-frozen using liquid nitrogen–cooled isopentane. Cross-sections (10 μm) were prepared using a cryostat (Leica CM1850) and stained with H&E or trichrome. Whole sections were imaged with the Hamamatsu NanoZoomer slide scanner, and the most representative sections are presented in this study. Other sections were immunolabeled overnight with antibodies against the dystrophin N-terminus (homemade, rabbit 246) (75), γ-sarcoglycan (NCL-g-SARC, Leica Biosystems), β-dystroglycan (NCL-b-DG, Leica Biosystems), periostin (ab1404150, Abcam), myosin heavy chains type I (BA-D5, DSHB), type IIa (SC-71, DSHB), type IIb (BF-F3, DSHB), or laminin 2 (L0663, rat, Sigma-Aldrich) diluted (1:100) in solutions containing Tris-buffered saline (TBS) with Tween 20 and 5% bovine serum albumin (BSA). Secondary antibodies goat anti-rabbit Alexa Fluor 790 (111-655-144, Jackson ImmunoResearch), goat anti-rabbit Alexa Fluor 488 (111-545-144, Jackson ImmunoResearch), goat anti-mouse IgG2a Alexa Fluor 488 (115-547-186, Jackson ImmunoResearch), goat anti-rabbit Alexa Fluor 594 mouse IgG2b (115-587-187, Jackson ImmunoResearch), goat anti-mouse IgG2b Alexa Fluor 350 (A21140, Invitrogen), goat anti-mouse IgM Alexa Fluor 488 (115-545-020, Jackson ImmunoResearch), goat anti-mouse IgG1 (115-587-185, Jackson ImmunoResearch), or goat anti-rat Alexa Fluor 594 (A11007, Invitrogen) were incubated for 2 hours diluted (1:100) in solutions containing TBS-Tween and 5% BSA. Slides were mounted using Immu-Mount (Epredia), and images were captured on a Nikon Eclipse 90i microscope. The myofiber size and minimal fiber diameter (miniFeret) were determined from laminin-positive sections. The percentage of dystrophin-positive myofibers was quantified using sections stained with anti-dystrophin and -laminin antibodies, while dystrophin-positive fiber type percentage was quantified from sections quadruply stained for dystrophin and myosin heavy chains. The fibrosis area was measured using sections stained with trichrome. Periostin area was quantified from sections stained with anti-periostin antibodies. Fiji image analysis software (version 2.0.0-rc-68/1.52g) (76) was used to quantify all the histology parameters cited above.

### Protein extraction, digestion, and peptide isobaric labeling.

Frozen gastrocnemius muscle tissue pieces (20–25 mg) were processed using a Percellys Cryolys Evolution bead beater (Bertin Technologies). Tissue samples were weighed in Percellys tissue homogenizing CKMix tubes (Bertin Technologies) and protein extraction buffer (7 M urea, 2 M thiourea, 0.4 M Tris pH 8.0, 20% [v/v] acetonitrile, 10 mM tris [2-carboxyethyl] phosphine [TCEP], 40 mM chloroacetamide, and 1 μL/100 μL buffer Pierce Universal Nuclease [Thermo Fisher Scientific]) was added at a ratio of 9 μL lysis buffer per mg of tissue. A 150 μL aliquot of each sample was transferred to a PCT tube with a 150 μL cap for the Barocycler NEP2320 (Pressure Biosciences, Inc.) and cycled between 35 kPSI for 20 seconds and 0 kPSI for 10 seconds, for 60 cycles at 37°C. After barocycling, the samples were centrifuged at 15,000*g* for 10 minutes. The samples were transferred to new 1.5 mL microfuge Eppendorf Protein LoBind tubes. Aliquots for each sample were taken for protein concentration determination by Bradford assay.

A bridged pooled normalizing sample was made for 2 TMTpro 16plex (Tandem Mass Tag, Thermo Fisher Scientific) experiments. The pooled sample was composed of equal-microgram aliquots of each gastrocnemius sample. An 18 μg aliquot of each sample and pooled sample was transferred to a new 1.5 mL Eppendorf Protein LoBind tube and brought to the same volume with extraction buffer. The samples were diluted 5-fold with LC-MS grade water. Next, trypsin (Promega) was added in a 1:40 ratio of trypsin to total protein. Samples were incubated at 37°C overnight, then were acidified with 0.3% (v/v) formic acid. Samples were cleaned using a MCX Stage tip (University of Minnesota Center for Metabolomics and Proteomics) and eluates were vacuum dried. Samples were resuspended with 0.1 M triethylammonium bicarbonate, pH 8.5, to a final protein concentration of 1 μg/μL.

For stable isotope labeling, a 14 μg aliquot for each sample was made and assigned a channel within a TMTpro 16plex. The samples were labeled with TMTpro 16plex isobaric label reagent in a 1:10 ratio of micrograms of protein to micrograms of TMTpro 16plex label according to the manufacturer’s instructions. Isobaric tag–labeled samples within the same experimental screen were multiplexed together into a new 1.5 mL Eppendorf tube, then vacuum dried and cleaned with a 1 mL SepPak C18 solid phase extraction cartridge (Waters Corporation). Each TMTpro 16plex sample was vacuum dried, resuspended in 20 mM ammonium formate, pH 10, 98% (v/v) water and 2% (v/v) acetonitrile, and fractionated offline by high-pH C18 reversed-phase chromatography as previously described (77). After fractionation, concatenated peptide fractions were C18 Stage tipped (78) and eluates were dried in vacuo.

### MS data acquisition.

Skeletal muscle TMTpro 16plex proteomics experiments were performed at the University of Minnesota in collaboration with the Center for Metabolomics and Proteomics (CMSP) departmental core facility. Gastrocnemius muscle was extracted from 5 experimental groups of mice, including male WT (C57BL/6), untreated *mdx*^4cv^ (B6Ros.Cg-Dmd*^mdx-4Cv^*/J), μDys, midi-Dys, and full-Dys mice. Mice treated with low-dose AAV gene therapy constructs were sacrificed 3 months after treatment, and untreated WT and *mdx*^4cv^ mice were age matched with treated mice. Each group consisted of *n* = 6 biological replicates for a total of 30 samples. Two TMTpro 16plex screens were run sequentially to include all samples split equally between each screen, along with a pooled normalization control sample included in each screen. Peptide pellets were resuspended in solution consisting of 95% water, 5% acetonitrile, and 0.1% formic acid. The peptide mixture was vortexed for 45 seconds and centrifuged for 2 minutes at 4,000*g*. Data were collected on a Thermo Fisher Scientific Orbitrap Eclipse mass spectrometer coupled to a Dionex Ultimate 3000 RSLCnano LC pump. Peptides from 17% (2 μL) of each concatenated set of fractions were separated using a 199-minute gradient at 0.315–0.325 μL/min with a 0–90% Buffer B gradient at a column temperature of 55°C on a C18-AQ ReproSil-Pur column measuring 400 mm with an internal diameter of 100 μm, 1.9 μm resin size, and 120 Å pore size (Dr. Maisch GmbH Ammerbuch). Buffer A consisted of water with 0.1% (v/v) formic acid and Buffer B consisted of acetonitrile with 0.1% (v/v) formic acid. High-field asymmetric-waveform ion mobility spectroscopy (FAIMS) was enabled during experimental acquisition with the following compensation voltage (CV) settings: –45 V, –60 V, and –75 V. Voltage was kept at 2.1 kV for positive-ion mode and the ion transfer tube temperature was set to 275°C. At the MS1 stage, the mass spectrometer scanned masses in the range of 400–1400 *m*/*z* at a resolution of 120K with an AGC target of 4.0 × 10^5^ over a 50-ms maximal injection time. At the MS2 stage, ions were fragmented by high-energy collisional dissociation (HCD) with a collision energy of 38% at a detector resolution of 50K with an AGC target of 1.25 × 10^5^ (250% relative to default) over a 150-ms maximal injection time, and the Fourier transform first mass mode was fixed at 110 *m/z*.

### Proteomics peptide spectrum matching and quantification.

Raw MS files were processed by CMSP in Proteome Discoverer v3.1 (Thermo Fisher Scientific). Peptide identification was performed by searching HCD MS/MS files against the UniProtKB/Swiss-Prot *Mus musculus* database (UP000000589; accessed August 18, 2023) appended with custom dystrophin sequences from AAV-Myo1 μDys5, midi-Dys, and full-Dys constructs. Database search files were merged with a common lab contaminant database (https://github.com/HaoGroup-ProtContLib) with the Sequest HT search engine and a 1% false discovery rate (FDR) was set for peptide-to-spectrum matches using the Percolator algorithm in Proteome Discoverer v3.1. The following parameters were used for spectral processing: MS1 tolerance of 20 ppm, MS2 tolerance of 0.08 Da, trypsin (full) digestion with a maximum of 2 missed cleavages, minimum peptide length of 6 and maximum peptide length of 50, with 10 maximum peptides reported. Cysteine carbamidomethylation was set as a static modification, while TMTpro lysine and N-terminal modifications, asparagine and glutamine deamidation, methionine oxidation, pyro-glutamic acid, N-terminal acetylation, methionine loss, and methionine loss with acetylation were set as dynamic modifications in Sequest. Only protein identifications with high FDR confidence (FDR < 1%) and containing 2 or more peptides were accepted. Reporter ion quantification was conducted using the TMTpro 16plex (lot YD372049) quantification method with a peak integration tolerance of 20 ppm and the most confident centroid method. Unique and razor peptides were used for quantification. All peptides were used for normalization and protein roll-up, and scaling was performed for inter-screen data normalization using a pooled average control sample. Hypothesis testing was performed using a 2-tailed *t* test (background based) for pairwise ratios. Grubbs’ test was used to identify and exclude single outlier data points.

### Western blot.

Proteins were extracted from gastrocnemius muscles using radioimmunoprecipitation analysis buffer (RIPA) supplemented with 1 mM PMSF and a 4% protease inhibitor cocktail (P8340, Sigma-Aldrich). Total protein concentration was determined using the Pierce BCA assay kit (Thermo Fisher Scientific). Samples were denatured at 100°C for 10 minutes, then 30 μg of protein lysates were separated in NuPage 4%–12% Bis-Tris polyacrylamide gels (Invitrogen) using 165 V for 1 hour at room temperature. Protein transfer to 0.45 μm PVDF membranes (Amersham Hybond) was performed at 120 V at 4°C for 2 hours. Membranes were blocked for 2 hours in TBS containing 5% nonfat dry milk and 0.005% Tween 20 before overnight incubation with antibodies against utrophin (from rabbit, gift from Froehner lab, University of Washington; ref. 79), dysferlin (Hamlet-CE, Leica Biosystems), γ-sarcoglycan (NCL-g-SARC, Leica Biosystems), or GAPDH (rabbit, G9545, Sigma-Aldrich) as a loading control. Secondary antibodies coupled to horseradish peroxidase were anti-mouse IgG2b (115-035-207, Jackson ImmunoResearch), anti-mouse IgG1 (115-035-205, Jackson ImmunoResearch), or goat anti-rabbit (111-035-144, Jackson ImmunoResearch). Blots were incubated for 2 hours at room temperature before visualization using Clarity Western ECL substrate (Bio-Rad) in the Chemidoc MP imaging system (Bio-Rad). The relative expression was determined by band densitometry measurements on unsaturated images using Fiji image analysis software.

### Data availability.

Source data to interpret, verify, and extend this research are provided in this paper. The MS proteomics data have been deposited to the ProteomeXchange Consortium via the PRIDE (80) partner repository with the dataset identifier PXD062324. Source data are provided in this paper. R script used to generate plots, filter, and analyze data is publicly available at: https://github.com/joh18358/Split-intein-mdx-proteomics Values for all data points in graphs are reported in the Supporting Data Values file.

### Statistics.

Comparisons between all experimental groups were performed using 1-way ANOVA with Tukey’s multiple-comparison correction. Scaled protein abundances were used to calculate pairwise fold changes based on the geometric means of all biological replicates from each sample group. Fold changes were calculated for pairwise comparisons between the following groups: *mdx*^4cv^/WT, AAVMYO1 μDys5-treated *mdx*^4cv^/WT, AAVMYO1 midi-Dys–treated *mdx*^4cv^/WT, AAVMYO1 full-Dys–treated *mdx*^4cv^/WT, *mdx*^4cv^/μDys5-treated *mdx*^4cv^, *mdx*^4cv^/midi-Dys–treated *mdx*^4cv^, *mdx*^4cv^/full-Dys–treated *mdx*^4cv^, μDys5-treated *mdx*^4cv^/midi-Dys–treated *mdx*^4cv^, μDys5-treated *mdx*^4cv^/full-Dys–treated *mdx*^4cv^, and midi-Dys–treated *mdx*^4cv^/full-Dys–treated *mdx*^4cv^. A 2-tailed, unpaired Student’s *t* test was used to calculate *P* values for pairwise fold changes, and the Benjamini-Hochberg method was used to control the FDR. Corrected *P* values were log-transformed and plotted against log-transformed fold change values to obtain volcano plots generated in R using the tidyverse package, and a minimum corrected *P*-value cutoff of 0.05 and minimum relative fold change cutoff of ±1 was applied to identify DEPs in pairwise comparisons. Comparisons between all experimental groups were performed using 1-way ANOVA with Tukey’s multiple-comparison correction. Full protein quantification datasets generated in Proteome Discoverer and lists of DEPs were imported to R for data filtering and visualization using the gplots, VennDiagram, and dplyr packages. Venn diagrams were used to obtain lists of overlapping and non-overlapping DEPs between distinct 2-group comparisons. Proteins with missing values for pooled samples in one or both screens were excluded from further analysis. Functional enrichment analysis was performed using Gorilla (81) and g:Profiler (82). For DEP gene ontology (GO) analysis, the target set included the DEP list and the background set the *Mus musculus* reference proteome. PCA plots were generated in R using the ggfortify package. One-way ANOVA statistical analysis and dataset filtering were performed in R. Bar graphs, GO enrichment visualizations, and heatmaps for DEPs of interest were performed in GraphPad Prism, version 10.2.

### Study approval.

All animal experiments were approved by the University of Washington’s Institutional Animal Care and Use Committee (IACUC).

## Author contributions

HT and JSC designed the AAV treatment and strategy. EEJ and JME conceptualized the proteomics study. HT produced and purified the AAV, injected mice, and collected muscle samples. EEJ prepared the muscle samples and proteins for proteomics analysis and performed the statistical and bioinformatic analysis. HT and TRR analyzed the muscle histology. HT and EEJ wrote the manuscript. JME and JSC provided reagents and edited the manuscript.

## Funding support

This work is the result of NIH funding, in whole or in part, and is subject to the NIH Public Access Policy. Through acceptance of this federal funding, the NIH has been given a right to make the work publicly available in PubMed Central.

- Muscular Dystrophy Association grant 1060372.
- Association Française Contre Les Myopathies (AFM-Telethon, grant 24777).
- NIH grants P30 DK017047 and P50 AR065139 to the Diabetes Research Center and the Wellstone Center, respectively, at the University of Washington.
- US Department of Defense grant MD220097.
- Bettencourt-Schueller Foundation fellowship (to HT).
- Philippe Foundation fellowship (to HT).
- Association Française Contre Les Myopathies (AFM-Telethon) fellowship (to HT).
- NIH Minnesota Muscle Training Grant 5T32AR007612 (to EEJ).
- NIH grant 5R01AR042423 (to JME).
- NIH High-end Instrumentation Grant S10OD028717 (support for the Orbitrap Eclipse instrumentation platform used for proteomics data acquisition).

## Supplementary Material

Supplemental data

Unedited blot and gel images

Supporting data values

## Figures and Tables

**Table 1:**
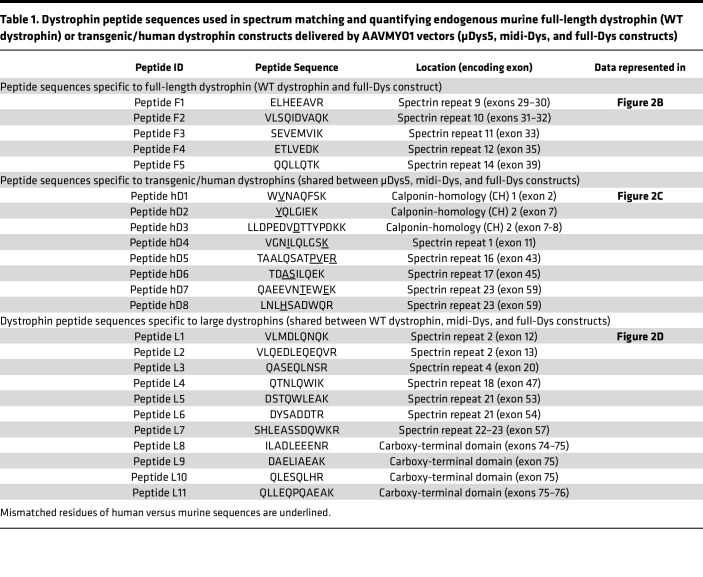
Dystrophin peptide sequences used in spectrum matching and quantifying endogenous murine full-length dystrophin (WT dystrophin) or transgenic/human dystrophin constructs delivered by AAVMYO1 vectors (μDys5, midi-Dys, and full-Dys constructs)

## Version 1 11/27/2025

In-Press Preview

## Version 2 01/23/2026

Electronic publication
