# Supplementary material for: Proteomics-based evaluation of AAV dystrophin gene therapy outcomes in mdx skeletal muscle
Source: JCI Insight. 2025 Nov 27;11(2):e197759. doi: 10.1172/jci.insight.197759 (PMC12892886; doi:10.1172/jci.insight.197759)
Supplement: Supplemental data [file jciinsight-11-197759-s155.pdf]

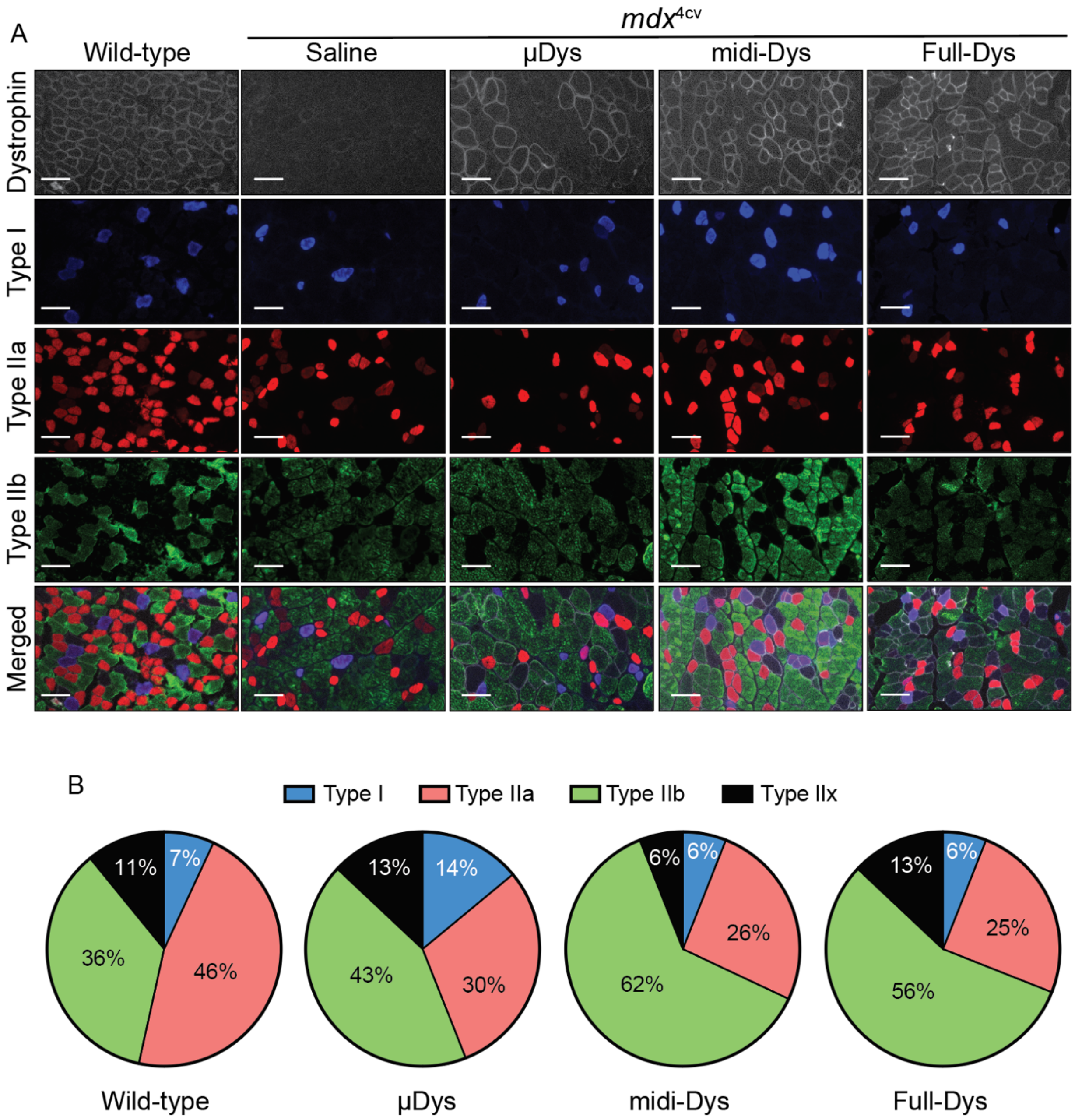

Supplementary Figure 1: A) Quadruple immunolabeling of gastrocnemius muscle cross-sections with antibodies specific to myosin heavy chains type I, IIa and IIb or dystrophin to correlate the dystrophin expression and fiber-type specificity (scale bars: 50µm). B) quantification of dystrophin-positive myofibers from the muscle sections stained with myosin heavy chains antibodies. ~1000 fibers were counted.

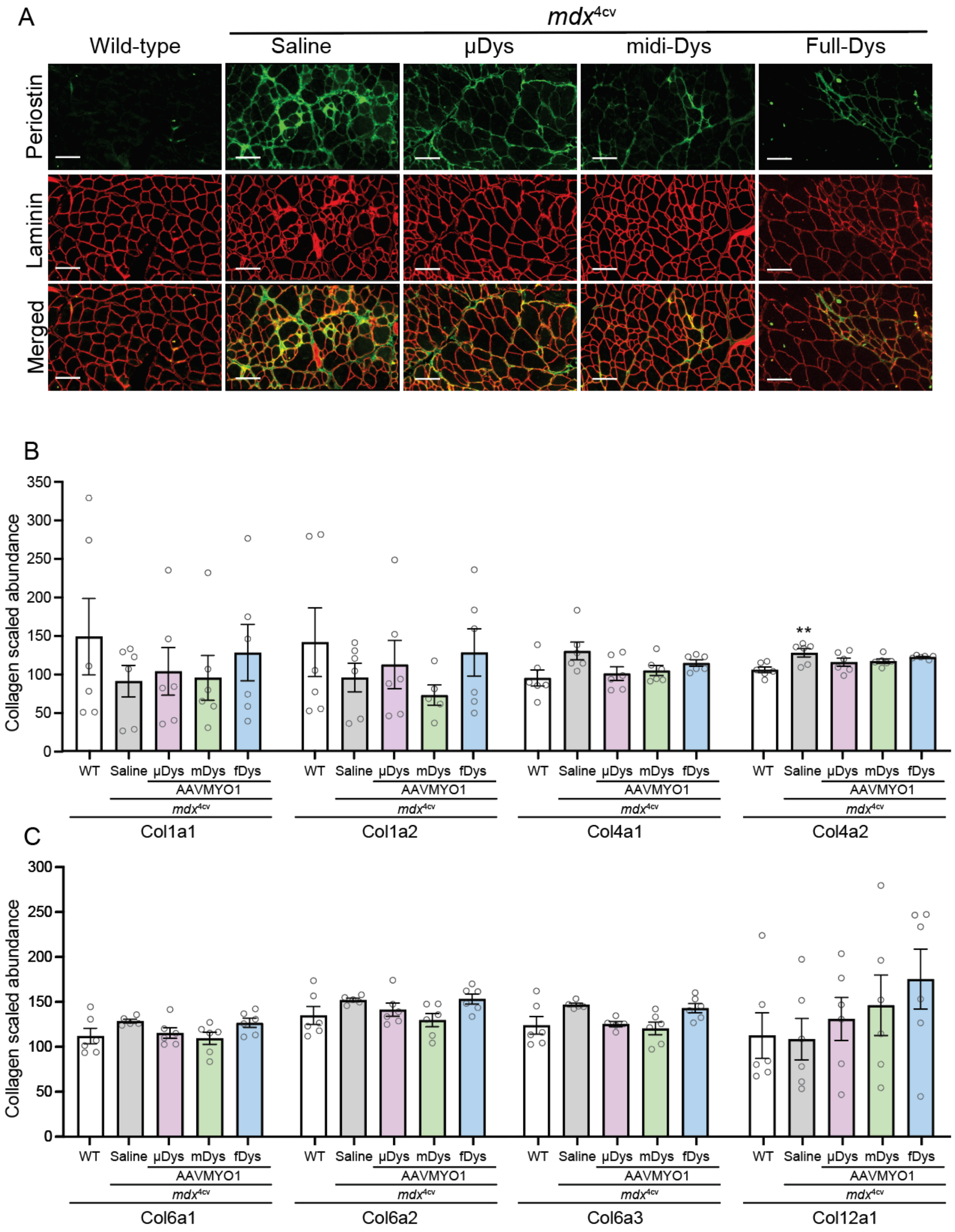
A
mdx4cv
Wild-type
Saline
μDys
midi-Dys
Full-Dys
Periostin
Laminin
Merged
B
Collagen scaled abundance
350
300
250
200
150
100
50
0
**
WT Saline μDys mDys fDys
AAVMYO1
mdx4cv
Col1a1
Col1a2
Col4a1
Col4a2
C
Collagen scaled abundance
300
250
200
150
100
50
0
WT Saline μDys mDys fDys
AAVMYO1
mdx4cv
Col6a1
Col6a2
Col6a3
Col12a1

Supplementary Figure 2: A) original panel presented in figure 3 showing periostin expression as well as laminin (scale bars: 50µm). B) and C) relative abundance of different collagen isoforms found to be unchanged in *mdx*^{4cv} mice at the age of 5 months, with the exception of Col4a2. Bar graphs depict means ± SEM from n=5-6 mice/group. Comparisons between groups were made using one-way ANOVA with Tukey's multiple comparisons test. $^{**}P<0.01$ *versus* the WT group. µDys: micro-dystrophin, mDys: midi-dystrophin, fDys: full-length dystrophin.

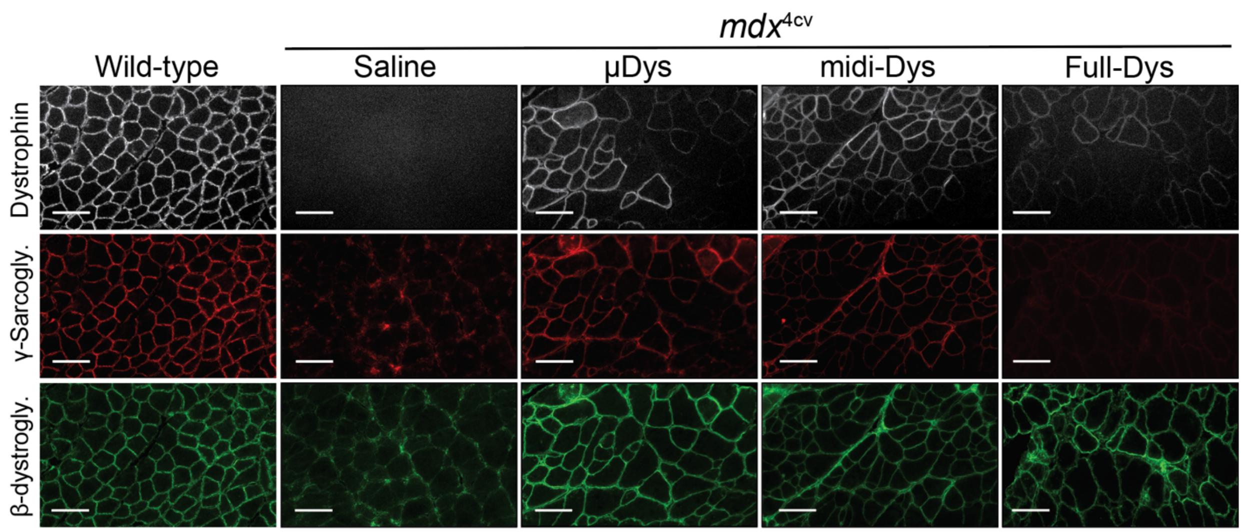

Supplementary Figure 3: Original panel presented in Figure 3 showing expression and localization of dystrophin and two DGC partners, γ-sarcoglycan and β-dystroglycan (scale bars: 100µm).

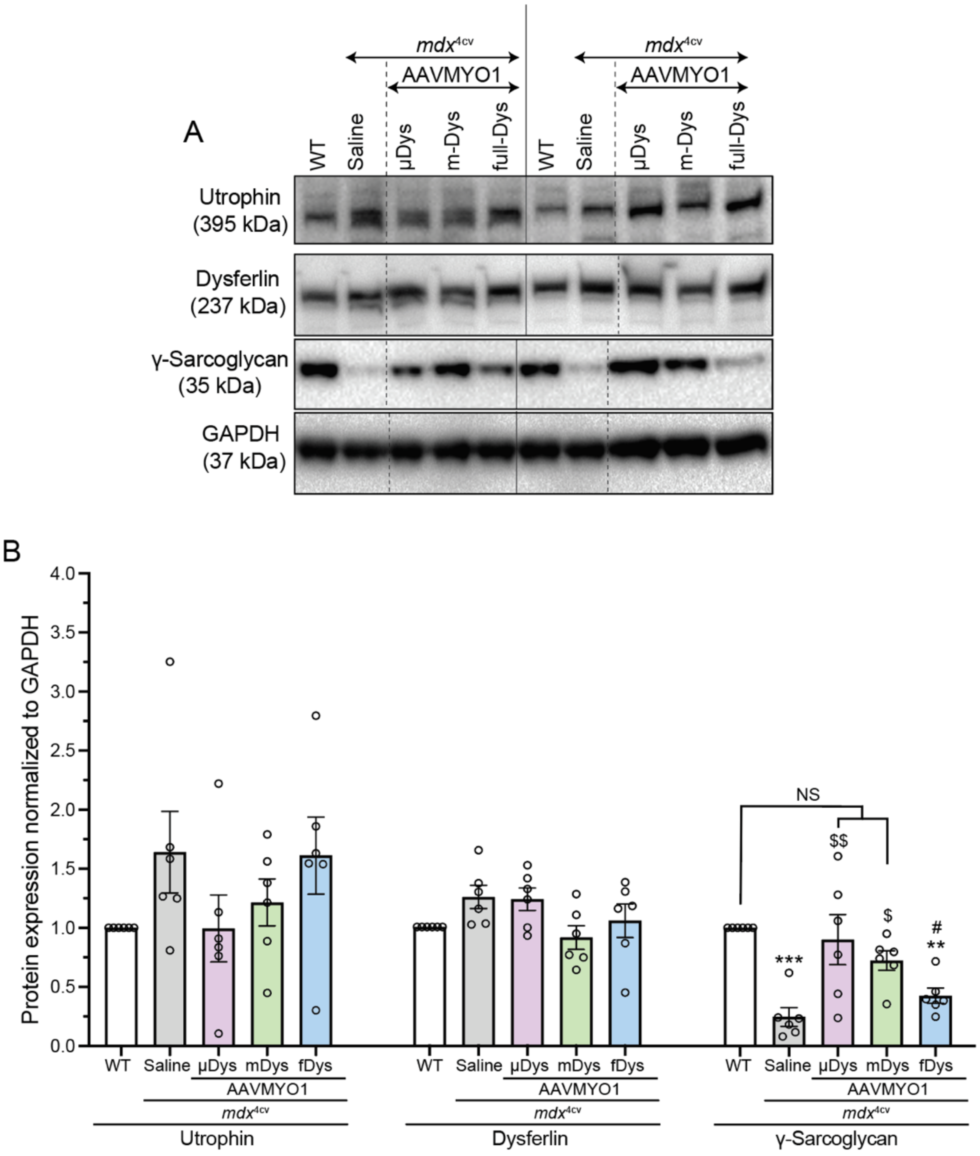

Supplementary Figure 4: Western blot validation of protein expression of key proteins. A) representative gel image of the same protein gastrocnemius samples stained with antibodies specific to utrophin, dysferlin and γ-sarcoglycan. B) Densitometry quantification of protein expression levels. Bar graphs depict means ± SEM from n=6 mice/group. NS: Not significant, $^{**}P<0.01$, $^{***}P<0.001$ *versus* WT; $^{\$}P<0.05$, $^{\$\$}P<0.01$ *versus* saline group; $^{\#}P<0.05$ *versus* µDys group using ANOVA test followed by Tukey's post hoc. µDys: micro-dystrophin, mDys: midi-dystrophin, fDys: full-length dystrophin.
