# Supplementary material for: Proteomics-based evaluation of AAV dystrophin gene therapy outcomes in mdx skeletal muscle
Source: JCI Insight. 2025 Nov 27;11(2):e197759. doi: 10.1172/jci.insight.197759 (PMC12892886; doi:10.1172/jci.insight.197759)

Uncropped western blots from Supplemental Figure 4A: Dashed rectangles indicate the approximate cropping used in the Supplemental Figure 4A. kDa: kilodalton

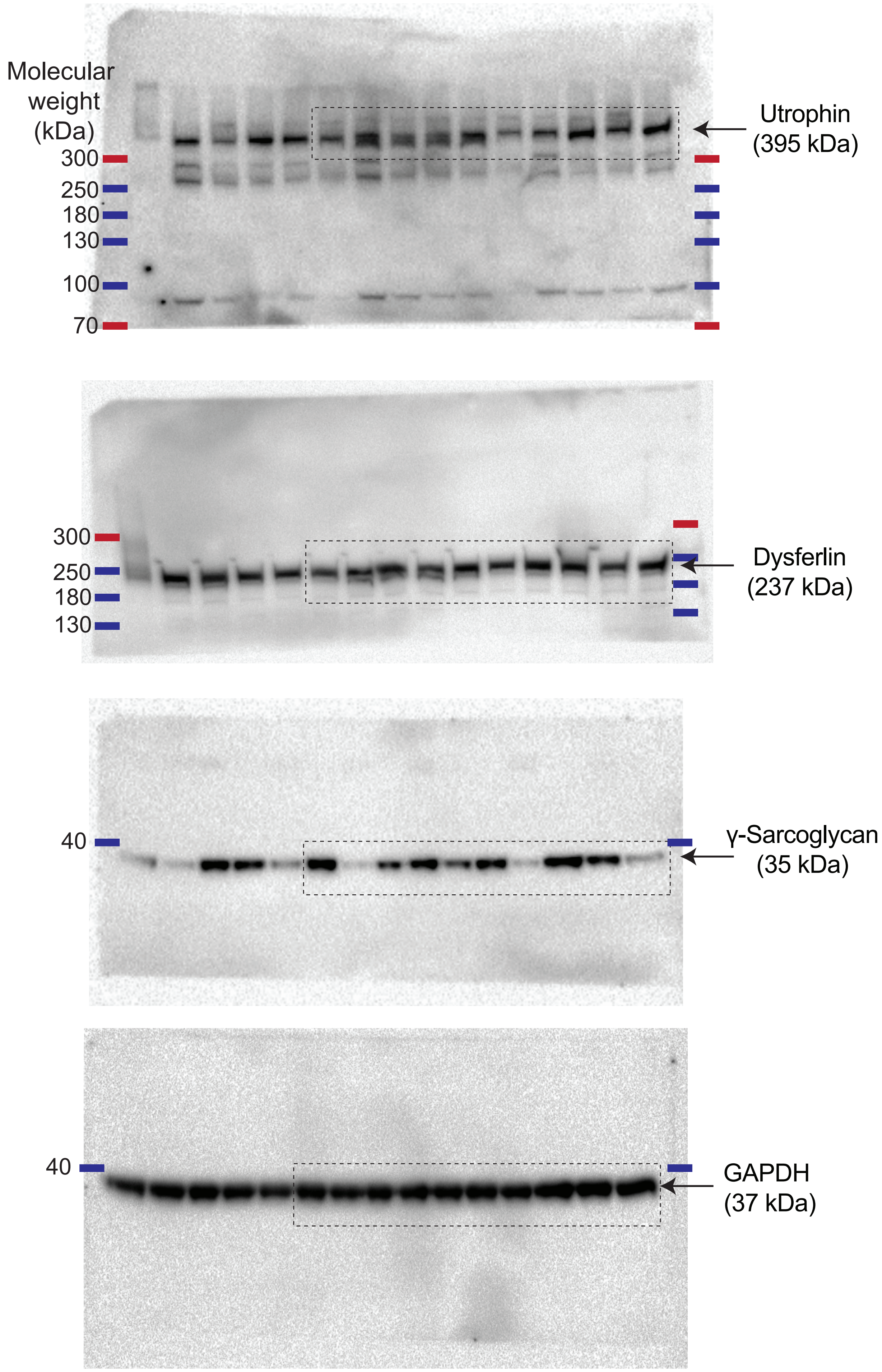

Supplement: Unedited blot and gel images [file jciinsight-11-197759-s156.pdf]
